# Prospective mental imagery as its link with anxiety and depression in prisoners

**DOI:** 10.1371/journal.pone.0191551

**Published:** 2018-03-15

**Authors:** Belén López-Pérez, Catherine Deeprose, Yaniv Hanoch

**Affiliations:** 1 Department of Psychology, Liverpool Hope University, Liverpool, United Kingdom; 2 School of Psychology, Plymouth University, Plymouth, United Kingdom; Brown University, UNITED STATES

## Abstract

Mental imagery is known to play a key role in the development and maintenance of depression and anxiety. Prisoners commonly experience psychological distress, but interventions to address this are currently lacking. We aimed to examine the link between prospective mental imagery and anxiety and depression among prisoners. One hundred twenty-three male prisoners from a Category C prison in southwest England participated in the study. They completed the Centre for Epidemiologic Studies Depression Scale (CES-D) and the General Anxiety Disorder Scale (GAD-7) to measure whether they experience depression and/or anxiety symptoms. Furthermore, they completed additional questionnaires to evaluate their prospective mental imagery. Results showed that 67.5% of prisoners presented with more depression symptoms and 27.7% with more anxiety symptoms. Supporting earlier findings, our data revealed that some dimensions of prospective mental imagery were significantly related with increased anxiety and depression symptoms in prisoners. Namely, intrusive negative personally relevant imagery was a positive predictor and likelihood of positive events a negative predictor of both anxiety and depression symptoms. The perceived likelihood of negative events was a positive predictor of depression. Intrusive verbal thought was a positive predictor of anxiety. The obtained results suggest the need to develop interventions not only targeting the reduction of prospective negative imagery but also the enhancement of positive mental imagery.

## Introduction

Eighty-two prisoners committed suicide in 2015 in England and Wales, representing the highest number of suicides in prisons in those countries in over 7 years [1]. As the prevalence of psychological disorders among prisoners is far higher than in the general population, the high rates of suicide in prison might not come as a surprise. Mental illness among prisoners, in fact, carries a heavy toll, as it has been linked to increased rates of recidivism [2], self-harm [3], and substance abuse [4] as well as reduced well-being [5]. Given the burden of depression for prisoners and prison authorities, gaining a better understanding of the mechanisms involved in prisoners’ mental illness has the potential to improve prevention and treatment programs and thus reduce rates of depression and anxiety.

Two disorders are particularly prevalent among prisoners: depression or major depression disorder, with prevalence rates of up to 65%, and general anxiety disorders, with prevalence rates of up to 37% [2, 5], representing a much higher prevalence than in the general population [6]. Both disorders have been linked to suicidal ideation in prisoners [7]. Thus, depression and anxiety need to be better understood within this at-risk population.

### Mental imagery in anxiety and depression

A large body of research has shown that depression and anxiety can develop or be maintained through cognitions, which can take the form of verbal thoughts and/or mental images [8]. Indeed, anxiety and depression have been linked to a cognitive bias towards negative material, which influences people’s responses to past, present, and future events [9]. Thus, mental images can trigger a range of emotional responses [10], as mental imagery is at the core of re-experiencing past events and imagining future (positive and negative) hypothetical events [11].

Although depression and anxiety often occur as comorbid disorders [12], they differ in terms of expectancies. While both are associated with negative expectancies, depression is also linked with reduced positive expectancies [13] and lower positive affect [12]. As imagery may trigger different emotional responses [14], researchers have investigated whether these different emotional responses and cognitions may be associated with differences in mental imagery [10].

To that aim, two approaches have been taken. The first approach has focused on the study of deliberate or intentional generation of positive and negative events by asking patients suffering from depression and anxiety to generate future and past experiences and evaluating whether these were positive or negative. In one of the first studies conducted, researchers found that participants with depression generated fewer positive experiences than controls, and participants with anxiety produced a greater number of negative experiences compared to controls [14]. In another study, anxious and anxious-depressed participants expected more negative experiences than controls and the anxious-depressed group also expected fewer positive experiences [15]. In a subsequent replication of these studies with non-clinical samples, it was found that only higher reports of anxiety symptoms but not depression symptoms was related to enhanced imagery for future negative events. Furthermore, only higher reports of depression symptoms were related with reduced imagery for positive events [16].

The second approach has explored intrusive or involuntary prospective mental imagery. Researchers have found an association between intrusive prospective visual images and mental disorders such as depression [17] and anxiety [18]. People with depression compared to controls tend to report more negative intrusive imagery [19], less positive imagery [20], and reduced vividness [21]. Intrusive negative imagery is also present in many anxiety disorders such as post-traumatic stress disorder, social phobia, and generalized anxiety disorder [22]. A study with patients diagnosed with depression or anxiety and matching controls has shown that patients with anxiety and depression reported less vivid positive imagery compared to controls. Patients with anxiety reported more vivid negative imagery compared to patients with depression and controls. Furthermore, both clinical groups reported more negative personal event imagery compared to controls [9]. Another study with patients suffering from depression and a matched control, reported that besides impoverished positive imagery (as highlighted in the previous study), patients with depression also exhibited excessive intrusive negative imagery and reduced vividness [21]. Taken together, research supports the idea that depression and anxiety are exhibit deficiencies in their imagery, and researchers have used mental imagery successfully to target intrusive thoughts to treat depression and anxiety [23, 24].

### The present research

Although there is a large body of research on the link between mental imagery and depression and anxiety symptoms [9] in the general population, the link between these variables among prisoners [5] is poorly understood. Given that the high prevalence of anxiety and depression among prisoners our study aims to address this gap in the literature.

We expected that both anxiety and depression would be negatively predicted by the perceived likelihood of generating positive scenarios, as previous research has shown that patients with depression or anxiety generate less positive imagery [9]. Furthermore, we predicted that both anxiety and depression would be positively predicted by the perceived likelihood of generating negative scenarios and by intrusive visual and verbal thought imagery, as previous research has found that patients in both clinical groups exhibited more negative and more intrusive imagery [19]. Finally, we included trait multi-modal imagery performance as a control variable.

## Method

The present study received ethical approval by the Ethics research committee from the School of Psychology at Plymouth University. Participants were free to consent and there were no incentives associated with participation. Capacity to consent was determined by allowing each prisoner to read a brief document containing the aims of the study and allowing them to return an opt-in form one week later if they wished to participate. Although some prisoners presented with mental disorders, none of the prisoners presented any cognitive impairment and their mental disorders did not affect their cognitive functioning; therefore, they were deemed suitable to provide consent for themselves.

### Participants

One hundred twenty-three male prisoners who were all detained in a Category C (medium security) prison in southwest England participated in the study. The data collection took place between 2014–2015. The ages of participants ranged from 20 to 77 years (*M* = 36.13 years, *SD* = 14.08). The prisoners were serving sentences of different lengths for a variety of crimes (30 for sexual offences against a child, 9 for violent offences, 11 for sexual offences against an adult, 12 for burglary, 6 for robbery, 5 for drug-related offences, 3 for murder, 2 for illegal possession of weapons, 1 for arson, 1 for breaching the official secrets act, and 1 for violating probation). For forty-two participants no offence was available. Information on the length of the sentence was available for only 40 participants (34 had a determinate sentence, 2 had a life sentence, and 4 an Imprisonment for Public Protection or IPP sentence). Given that sentence length was not available for all participants, and that only six participants had an indeterminate sentence, we excluded sentence length from the analysis. Of the 81 prisoners whose mental health data were available, 58 were not diagnosed with any mental disorder at the time of the study, 15 had been diagnosed with depression, 3 with schizophrenia, 1 with autism, and 1 with bipolar disorder. One participant had been assessed as having a high level of psychopathic traits, and 2 participants had other mental health problems.

### Measures

**Center for Epidemiologic Studies Depression** (**CES-D) Scale.** The CES-D Scale [20] is a 20-item instrument aimed at determining participants’ self-reported symptoms of depression within the last week on a 4-point Likert scale ranging from 1 (*rarely or none of the time*/*less than 1 day*) to 4 (*most or all of the time/5–7 days*): for example, “I felt my life had been a failure” (α = .90). Although scores of 16 or above indicate that individual may present depressive symptoms, this may not be used as a diagnostic tool on its own.

**General Anxiety Disorder Scale** (**GAD-7).** The GAD-7 [21] is a 7-item questionnaire that measures the presence of generalized anxiety disorder symptoms through assessing the frequency of each possible symptom on a 4-point Likert scale ranging from 0 (*not at all*) to 3 (*nearly every day*): for example, “not being able to stop or control worrying” (α = .92). The boundaries for severity are 5, 10, and 15 for mild, moderate, and severe anxiety symptoms, respectively. As with the CES-D this may not be considered as a diagnostic tool on its own.

**Impact of Future Events Scale–Negative (IFES***-***N).** The IFES-N [19] is a questionnaire that assesses the impact of intrusive potential future events and personally relevant imagery. First, participants are asked to identify one *negative* future event that they had been imagining over the past 7 days. The rest of the questionnaire comprises 22 items answered on a 5-point Likert scale ranging from 0 (*not at all*) to 4 (*extremely*): for example, “I believed my thoughts about the future would definitely happen and would become real” (α = .84). A higher score indicates a greater level of prospective intrusive negative imagery. We did not include the two last items (i.e., I felt energetic and excitable and I felt elated and optimistic) as they refer to a positive emotional state and may only be relatable to positive imagery.

**Intrusive Visual Imagery** **Questionnaire.** The Intrusive Visual Imagery Questionnaire [22] is a 10-item questionnaire that evaluates the extent to which participants experience intrusive visual imagery on a 5-point Likert scale ranging from 1 (*strongly disagree*) to 5 (*strongly agree*): for example, “there are images that come to mind that I cannot erase” (α = .90).

**Intrusive Verbal Thought** **Questionnaire.** The Intrusive Verbal Thought Questionnaire [22] is a 10-item questionnaire that assesses participants’ tendency to experience intrusive thoughts on a 5-point Likert scale ranging from 1 (*strongly disagree*) to 5 (*strongly agree*): for example, “there are verbal thoughts that come to mind that I cannot erase” (α = .92).

**Prospective Imagery Task** (**PIT).** The PIT [23] requires participants to generate a mental image in response to 30 statements about specific negative scenarios (e.g., “you will have a serious disagreement with a good friend”) or positive future scenarios (e.g., “you will make good and lasting friendships”). Participants are then asked to rate on a 5-point Likert scale how vivid the imagery is (1: *no image at all*, to 5: *more vivid than reality*), how likely the imagined scenario is to occur (1: *not at all likely to occur*, to 5: *extremely likely to occur*), and how well they can imagine experiencing the event (1, *not at all*, to 5, *completely*) for both types of scenarios. Thus, the questionnaire yields six total scores: positive vividness items (α = .76); positive likelihood items (α = .82); positive experience items, (α = .79); negative vividness items (α = .80); negative likelihood items (α = .76); and negative experience items (α = .81). This task was administered with modifications introduced in previous research [25].

**Plymouth Sensory Imagery Questionnaire** (**PSIQ).** The PSIQ [24] is a 35-item questionnaire that assesses trait imagery in the modalities of vision (e.g., “imagine the appearance of a friend you know well”; α = .72), sound (e.g., “imagine the sound of an ambulance siren”; α = .86), smell (e.g., “imagine the smell of burning wood; α = .50), taste (e.g., “imagine the taste of mustard; α = .77), touch (e.g., “imagine touching warm sand; α = .86), bodily sensation (e.g., “imagine the bodily sensation of relaxing in a warm bath; α = .76), and emotion (e.g., “imagine feeling excited; α = .77) on an 11-point Likert scale ranging from 0 (*no image at all*) to 10 (*image as clear and vivid as real life*). We averaged the different subscales to create an index of trait multi-modal imagery.

#### Procedure

Every prisoner in the prison at the time of the study was asked to participate (around 500). Participants were free to consent and there were no incentives associated with participation. Of those approached, 123 agreed to participate. Capacity to consent was determined by allowing each prisoner to read a brief document containing the aims of the study and allowing them to return an opt-in form one week later if they wished to participate. Although some prisoners presented with mental disorders, none of the prisoners presented any cognitive impairment and their mental disorders did not impact their cognitive functioning; therefore, they were deemed suitable to provide consent for themselves. The study took place in a large room within the prison used for treatment groups. Each participant was tested individually and a research assistant was always in the room to answer any questions. After signing the consent form, each participant completed the different questionnaires in a randomized order. After the participant completed all the questionnaires, a research assistant checked the questionnaire booklet to make sure there were not any answers left blank. Finally, participants were debriefed.

## Results

### Clinical characteristics

The data for this study is available in supplementary material [S1 Data]. The scores on the CES-D ranged from 0 to 62 (*M* = 21.11, *SD* = 12.99) and the scores on the GAD-7 ranged from 0 to 21 (*M* = 7.64, *SD* = 6.27). From the overall sample, 83 participants (67.5%) scored above the cut-off points for presenting with depression symptoms, 34 (27.7%) with mild anxiety, 23 (16%) with moderate anxiety, and 19 (15.4%) with severe anxiety symptoms (Table 1). Twenty-one participants (17%) presented with both anxiety and depression. The scores on depression and anxiety symptoms were highly correlated (*r* = .61, *p* = .001).

**Table 1:** Means and standard deviations of main variables in the study.

Variable	*M*	*SD*
Trait multi-modal imagery	35.77	8.17
Intrusive visual imagery	32.94	10.07
Intrusive verbal thought	27.69	10.22
IFES-N	35.43	16.24
VNS	34.64	11.97
ENS	31.23	9.36
LNS	33.29	7.44
VPS	29.34	8.37
EPS	27.38	8.00
LPS	29.55	7.33

*Note*. IFES-N = Impact of Future Events Scale–Negative; VNS = vividness of negative scenarios; ENS = experience of negative scenarios; LNS = perceived likelihood of negative scenarios; VPS = vividness of positive scenarios; EPS = experience of positive scenarios; LPS = perceived likelihood of positive scenarios.

### Mental imagery in prisoners

Before running any analysis, we checked for possible multivariate outliers by using the Mahalanobis distance [26]. After calculating this for all the key variables in the study, six participants were deleted. In regard to intrusive imagery, prisoners’ ratings for visual imagery were significantly higher than their ratings for verbal imagery, *t*(122) = 7.87, *p* = .001, confidence interval 95% (CI) [3.92, 6.56], *d* = 2.09. Finally, in relation to vividness, likelihood, and experience of positive and negative scenarios, the results showed that prisoners reported higher vividness, *t*(122) = -4.66, *p* = .001, CI [-7.52, -3.04] *d* = 1.18, experience, *t*(122) = -4.31, *p* = .001, CI [-5.66, -2.10] *d* = 1.01., and likelihood, *t*(122) = -3.73, *p* = .001, CI [-5.71, -1.75] *d* = 1.09, for negative scenarios compared to positive ones (Table 1).

### Mental imagery and its link with prisoners’ anxiety and depression

First we ran a correlation analysis between the scores of depression and anxiety and the scores on the different visual imagery scales. Results showed that trait multi-modal imagery, vividness of positive scenarios, perceived likelihood of positive scenarios, and experience of positive scenarios were negatively related to both, anxiety and depression (Table 2). Furthermore, IFES-N, intrusive verbal thought, intrusive visual imagery, and perceived likelihood of negative scenarios were positively related to depression and anxiety (Table 2). Given that the number of prisoners with depression and anxiety was not equivalent to the number of prisoners without any mental disorder, we decided to use the scores of depression and anxiety symptoms as dependent variables in a regression analysis rather than comparing prisoners with and without depression and anxiety as a grouping variable.

**Table 2:** Correlations between measures the scores of depression and anxiety and the different imagery variables in the study.

	Trait multi-modal Imagery	IFES-N	IVI	IVT	VPS	VNS	LPS	LNS	EPS	ENS
Depression Scores	-.30**	.65**	.48**	.43**	-.29**	.18*	-.44**	.37**	-.34**	.24**
Anxiety Scores	-.29**	.52**	.37**	.44**	-.26**	.04	-.33**	.28**	-.20*	.17

*Note*. IFES-N = Impact of Future Events Scale–Negative; IVI = Intrusive Visual Imagery; IVT = Intrusive Verbal Thought; VNS = vividness of negative scenarios; ENS = experience of negative scenarios; LNS = perceived likelihood of negative scenarios; VPS = vividness of positive scenarios; EPS = experience of positive scenarios; LPS = perceived likelihood of positive scenarios; EPS = experiencing positive scenarios; and ENS = experiencing Negative Scenarios;

*p < .05;

** *p* < .01.

Given that anxiety and depression symptoms are co-morbid [12], we run two separate regression analyses entering the scores in the CES-D and GAD-7 as dependent variables, respectively. In each regression analysis, we entered the scores on the different imagery questionnaires and age as predictors. For depression symptoms there were no multicollinearity between the variables and the zero-order correlations between the variables were below .80, the tolerance was higher than .01, and the variance inflation factor (VIF) was lower than 10 [27] (Table 3). Results of the regression analysis showed that intrusive imagery as assessed through the IFES-N was a positive predictor of prisoners’ depression symptoms, whereas the perceived likelihood and vividness of positive scenarios were negative predictors (Table 3).

**Table 3:** Results of linear regression analysis predicting depression.

Predictor	*B*	*SE*	β	*t*	*Zero-order correlations*
R^2^ = .58					
Age	.02	.06	.02	.26	-.07
Trait multi modal	-.21	.12	-.13	-1.76	-.29
Intrusive visual imagery	.08	.14	.06	.53	.46
Intrusive verbal thoughts	.01	.13	.01	.05	.36
Vividness positive scenarios	-.69	.24	-.44**	-2.91	-.27
Vividness negative scenarios	-.22	.14	-.19	-1.58	.18
Likelihood positive scenarios	-1.01	.29	-.53**	-3.45	-.43
Likelihood negative scenarios	.19	.22	.11	.87	.31
Experiencing positive scenarios	-.31	.24	-.19	-1.28	-.34
Experiencing negative scenarios	.23	.20	.17	1.16	.29
IFES-N	.39	.07	.48**	5.88	.63

*Note*. IFES-N = Impact of Future Events Scale–Negative;

*p < .05;

** *p* < .01.

For anxiety symptoms, there were not any multicollinearity between the variables (Table 4). Results of the linear regression showed that intrusive imagery as assessed through the IFES-N and intrusive verbal thought were positive predictors of prisoners’ anxiety symptoms, whereas the perceived likelihood of positive scenarios was a negative predictor (Table 4).

**Table 4:** Results of linear regression analysis predicting anxiety.

Predictor	*B*	*SE*	β	*t*	*Zero-order correlations*
R^2^ = .41					
Age	.01	.04	.03	.35	.001
Trait multi modal	-.08	.07	-.09	-1.10	-.24
Intrusive visual imagery	-.11	.08	-.17	-1.35	.35
Intrusive verbal thoughts	.17	.07	.28	2.32*	.43
Vividness positive scenarios	.12	.14	.16	.89	-.26
Vividness negative scenarios	-.14	.08	-.25	-1.73	.04
Likelihood positive scenarios	-.37	.17	-.40	-2.17*	-.29
Likelihood negative scenarios	.21	.13	.25	1.62	.26
Experiencing positive scenarios	.06	.14	.08	.44	-.19
Experiencing negative scenarios	.03	.12	.04	.24	.26
IFES-N	.16	.04	.39	4.05**	.52

*Note*. IFES-N = Impact of Future Events Scale–Negative;

*p < .05;

** *p* < .01.

## Discussion

There are over 2 million prisoners in the United States and over 84,000 in the United Kingdom. With rates of depression and anxiety at 65% and 37%, close to 1.5 million prisoners are affected by depression and close to a million from anxiety. Given the economic, social, and health ramifications associated with prisoners’ mental health problems, a better understanding of the underlying mechanisms involved in these mental disorders could help practitioners design prevention and treatment programs. The present study is the first, to our knowledge, to explore the link between the production of mental imagery and depression/anxiety among prisoners.

Previous literature has shown that mental imagery is an important factor in the maintenance of anxiety and depression [15]. People with anxiety and depression have reported less vivid positive imagery and more vivid negative imagery compared to controls [9]. Given that the mental health problems of prisoners can have a serious negative impact on a range of behaviours (recidivism, drug abuse, suicide) [2], it is important to examine the factors that can be targeted through interventions. In the present study, a large proportion of prisoners presented with depression or anxiety symptoms, which fits earlier findings [6]. Furthermore, 17% of the prisoners presented symptoms of both disorders, a high rate of comorbidity [28].

Results from the regression analyses indicated that both self-reported depression and anxiety symptoms were positively predicted by intrusive negative imagery in offenders, as assessed with the IFES-N. This is in line with previous research that found patients with depression and anxiety reported higher intrusive negative personally relevant imagery compared to controls [9]. Although intrusive negative imagery has been described as a symptom of anxiety [22], it is only recently that researchers have found that intrusive negative imagery is linked to depression [29].

The likelihood of experiencing positive scenarios was a negative predictor of both depression and anxiety symptoms. This is also in line with earlier results, which found that patients with depression and anxiety rated the likelihood that positive events will take place as less probable than controls [9]. Furthermore, previous research has also shown that a lower perception of likelihood of occurrence of positive events was linked to higher hopelessness [30]. In fact, prisoners have been depicted as exhibiting higher hopelessness [31] and prisoners’ mental health vulnerability has been linked with this variable [32]. Thus, intervention programs could target prisoners’ perception of the likelihood of positive events occurring, as it may serve as a protective factor against anxiety and depression.

Finally, intrusive verbal thought was a positive predictor of self-reported anxiety symptoms in prisoners. This result is in line with previous research that found individuals with anxiety tended to report intrusive verbal thoughts [33]. In fact, in generalized anxiety disorder, worries usually take the form of verbal thoughts [34] and hence therapy is focused on encouraging individuals to generate images rather than verbal prompts of what worries to them [22].

Although the present study has produced novel and important results, it does have a number of limitations. First, the limited number of prisoners without any mental health problems prevented us from comparing prisoners with and without depression and anxiety. Second, we did not have a control non-offending group, which limits the ability to generalize our finding to other populations. Future research may consider comparing offenders that may present with anxiety and depression to offenders without mental disorders, clinical outpatients, as well as non-offending population without mental issues. This may help to disentangle whether the results are also driven by the prison environment. Second, anxiety and depression symptoms were assessed by only a single self-report measure. Future research should consider the use of different measures (e.g., diagnostic interviews) to establish whether prisoners present with depression or anxiety in order to run comparisons with a matching control group. Furthermore, future research may benefit from a longitudinal perspective in order to establish a possible causal relation between anxiety and depression and mental imagery in prisoners by comparing prisoners who were incarcerated recently and those who have been in prison longer periods of time (e.g. a year and over). That way, this could help to determine the contributing factor of the prison environment and the role of mental imagery in their development and maintenance. Finally, we did not have data regarding length of sentence and length of time in prison for some of the participants. Given that these two variables may affect prisoners’ mental health; they should be controlled for in future studies.

Despite these limitations, the present findings suggest that mental imagery is linked with depression and anxiety symptoms in offenders. Although it is premature to suggest changes in the current existing intervention programs based on Cognitive Behavioral Therapy [35], the obtained results indicate the need to study further mental imagery and consider its potential to aid current interventions in prison.

## Supporting information

S1 Data This file contains all the data. (SAV)
