# Correction: Prospective mental imagery as its link with anxiety and depression in prisoners

**DOI:** 10.1371/journal.pone.0195929

**Published:** 2018-04-11

**Authors:** Belén López-Pérez, Catherine Deeprose, Yaniv Hanoch

In the title of this article, the word “as” should be “and”. The correct title is: Prospective mental imagery and its link with anxiety and depression in prisoners. The correct citation is: López-Pérez B, Deeprose C, Hanoch Y (2018) Prospective mental imagery and its link with anxiety and depression in prisoners. PLoS ONE 13(3): e0191551. https://doi.org/10.1371/journal.pone.0191551
